# Ponderomotive forces in the system of two nanoparticles

**DOI:** 10.1038/s41598-022-22510-8

**Published:** 2022-10-22

**Authors:** Valeri Lozovski, Volodymyr Lysenko, Natalia Rusinchuk

**Affiliations:** 1 Institute of High Technologies, Taras Shevchenko National University of Kyiv, Volodymyrska Str. 60, Kyiv, 01602 Ukraine; 2 Lashkariov Institute of Semiconductor Physics, National Academy of Sciences of Ukraine, Nauki Ave. 45, Kyiv, 02000 Ukraine

**Keywords:** Nanoscience and technology, Nanoparticles

## Abstract

Mechanical consequences of electromagnetic interaction of two nanoparticles have been modeled and simulated. It has been shown that the local field enhancement effect in the studied system causes the appearance of the local field gradients. As a consequence, the local field gradients can lead to ponderomotive forces acting on the nanoparticles near their surface. In the work, the model describing the phenomena has been developed. The model is based on the near-field interaction in the self-consistent system and the effective susceptibility concept. Using the model distribution of the local field in the system of two different-sized nanoparticles has been calculated and ponderomotive forces directions and values were simulated. It has been shown that in the system of two different-sized nanoparticles the forces act mainly on the surface of the bigger nanoparticle and for some systems, the value of its density per volume unit may acquire up to several tens of nano newtons. Possible application of the results to the study of biological systems has been also discussed.

## Introduction

A system of two nanoparticles is the simplest nano-sized system, which is used for understanding the processes occurring at the nano-scale. In such a system the interactions can be described analytically, which is needed for analysis of the system properties caused by the interactions inside it. Interaction between two nanoparticles leads to the adsorption of one particle to another one due to Van der Waals or Casimir forces^1,2^. This interaction can have both attractive and repulsive parts caused by the nonlinear polarizability of small particles^3,4^. The sign of the force can be changed from attractive to repulsive by a suitable choice of interacting materials immersed in a fluid as it was discussed in^5^. Moreover, it was demonstrated that self-consistent electrodynamical interaction between two different-sized nanoparticles can lead to the repulsive part in adsorption potential^6^. The forces may also become repulsive for a special system geometry^7,8^ or interacting magnetic bodies^9^. Anyway, the forces lead to the formation of the stable system of two nanoparticles which under the action of external electromagnetic radiations (it can be daylight, for example) usually demonstrate the local field enhancement effect^10–12^. Then, close to the so-called ‘hot spots’ (areas with high local field values) rather strong gradients of the local field can be observed^13,14^. The gradients, in turn, are the cause of the so-called ponderomotive forces which act on the nanoparticle. The review devoted to the acting of ponderomotive forces on the different systems was shown in^15,16^. One should note that the problem of ponderomotive forces in the systems of nanoparticles was considered in numerous works. For example, it was shown that the optical binding of the asymmetric system of two nanoparticles can lead to the occurrence of the forces applied to the center of mass of the system^17^. The biological applications of optical forces caused by inhomogeneities of the local field were discussed in Ref.^18^ The action of the force between two or more particles exposed to nonresonant light is considered in^19^. Force is one of the key tools for manipulation of the particles by light.

If the polarization is distributed over the surface of the particle (which can be observed, for example, in the colloidal solutions of nanoparticles), the ponderomotive forces caused by local field gradients acting on the dipoles occur. Development of the model for self-consistent description and calculation of the forces connected with such electromagnetic interaction is the main aim of this work.

## The methods and main equations

Consider the system of two different-sized nanoparticles. The bigger nanoparticle consists of a core and a shell. The system symmetry axis OZ connects the centers of the particles. Suppose the external light incidence to the system. To demonstrate the effect of the interparticle interaction we need to calculate the effective susceptibility^20^ of the system under consideration. It is convenient to use the pseudovacuum Green function method^21^. Consider the medium consisting of the environment in which the small nanoparticle is embedded. Let the effective susceptibility of the small nanoparticle is ${\rm X}_{ij}^{(p)} ({\mathbf{R}},\omega )$. Note, the effective susceptibility is the characteristic of the object and depends on the material of which the object is fabricated and the dimension and shape of the object. Effective susceptibility connects the linear response (polarization) to the external field, namely $P_{i} ({\mathbf{R}},\omega ) = \varepsilon_{0} {\rm X}_{ij}^{(p)} ({\mathbf{R}},\omega )E_{j}^{(0)} ({\mathbf{R}},\omega )$^20^. Then, calculation of effective susceptibility provides the taking into account the self-action processes.

The electrodynamic Green function of the environment is $G_{ij}^{(0)} ({\mathbf{R}},{\mathbf{R}}^{^{\prime}} ,\omega )$. Then, using the method proposed in^16^, the pseudovacuum Green function $G_{ij}^{(m)} ({\mathbf{R}},{\mathbf{R}}^{^{\prime}} ,\omega )$ of the system ‘small nanoparticle-environment’ can be written as follows (see, Appendix)

1 $$\begin{aligned} G_{ij}^{(m)} ({\mathbf{R}},{\mathbf{R^{\prime}}},\omega ) & = G_{ij}^{(0)} ({\mathbf{R}},{\mathbf{R}}^{^{\prime}} ,\omega ) + G_{ij}^{(R)} ({\mathbf{R}},{\mathbf{R}}^{^{\prime}} ,\omega ) \\ & = G_{ij}^{(0)} ({\mathbf{R}},{\mathbf{R}}^{^{\prime}} ,\omega ) - k_{0}^{2} \int\limits_{{V_{p} }} {d{\mathbf{R}}^{^{\prime\prime}} G_{ik}^{(0)} ({\mathbf{R}},{\mathbf{R}}^{^{\prime\prime}} ,\omega ){\rm X}_{kl}^{(p)} ({\mathbf{R}}^{^{\prime\prime}} ,\omega )G_{lj}^{(0)} ({\mathbf{R}}^{^{\prime\prime}} ,{\mathbf{R}}^{^{\prime}} ,\omega )} , \\ \end{aligned}$$

where the integration is over the small nanoparticle volume, $k_{0}^{{}} = \omega /c$, *c* is the speed of light, and lower indexes mean x, y, and z in the Cartesian coordinate system. Here and below, we use the Einstein notation summation, meaning that $A_{ij} B_{jl} = \sum\limits_{j = x,y,z} {A_{ij} B_{jl} = A_{ix} B_{xl} + A_{iy} B_{yl} + A_{iz} B_{zl} }$. In Eq. (1) $G_{ij}^{(R)} ({\mathbf{R}},{\mathbf{R}}^{^{\prime}} ,\omega )$ is the impact of the smaller nanoparticle on the pseudovacuum Green function due to the local field redistribution.

**Figure 1:**
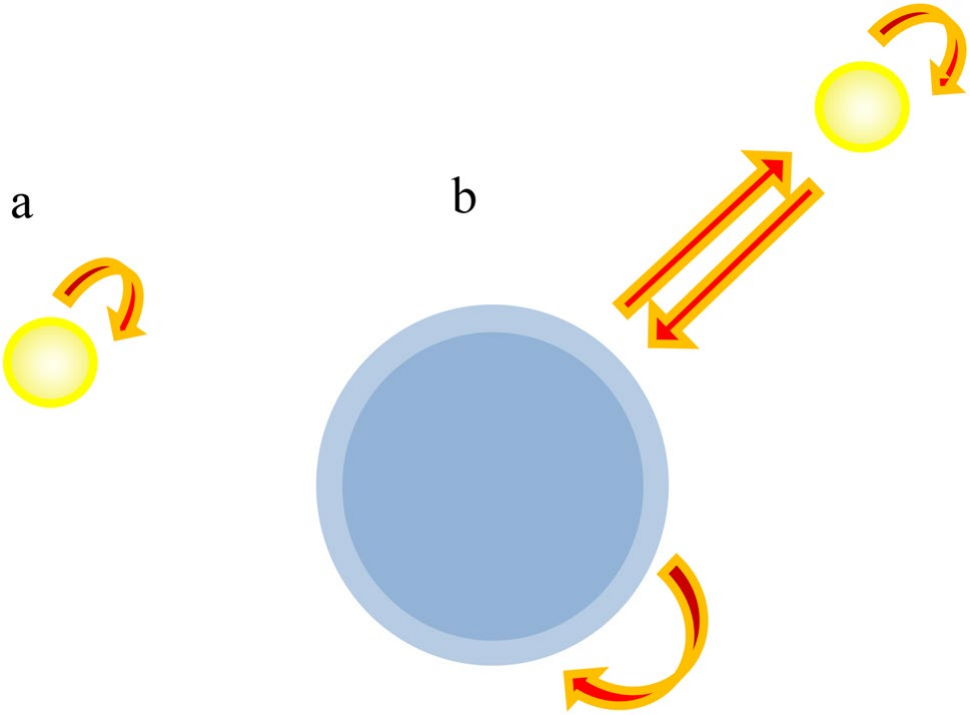
Scheme of self-action processes taken into ac-count when the effective susceptibilities ${\rm X}_{ij}^{(b)} ({\mathbf{R}},\omega )$ and ${\rm X}_{ij}^{(p)} ({\mathbf{R}},\omega )$—(**a**); and $\Xi_{ij}^{(b)} ({\mathbf{R}},\omega )$—(**b**).

In the frame of effective susceptibility concept, developed in^20^, the effective susceptibility of the big nanoparticle embedded in the pseudovacuum (the ‘new’ medium consisting of an environment and small nanoparticle) described by the Green function $G_{ij}^{(m)} ({\mathbf{R}},{\mathbf{R^{\prime}}},\omega )$ can be calculated according to

2 $$\Xi_{ij}^{(b)} ({\mathbf{R}},\omega ) = \left[ {\left( {{\rm X}_{ij}^{(b)} ({\mathbf{R}},\omega )} \right)^{ - 1} - k_{0}^{2} \int\limits_{{V_{v} }} {d{\mathbf{R}}^{^{\prime}} G_{ji}^{(R)} ({\mathbf{R}},{\mathbf{R}}^{^{\prime}} ,\omega )} } \right]^{ - 1} ,$$

where ${\rm X}_{ij}^{(b)} ({\mathbf{R}},\omega )$ is the susceptibility of the big nanoparticle which can be evaluated from the following equation^22,23^, which may be applied only for spherical nanoparticles with shells:

3 $${\rm X}_{ij}^{(b)} ({\mathbf{R}},\omega ) = \delta_{ij} {\rm X}^{(b)} (\omega ), \, {\rm X}^{(b)} (\omega ) = 3\;\frac{{(\varepsilon_{2} - \varepsilon_{m} )(\varepsilon_{1} + 2\varepsilon_{2} ) + f_{1} (\varepsilon_{1} - \varepsilon_{2} )(\varepsilon_{m} + 2\varepsilon_{2} )}}{{(\varepsilon_{2} + 2\varepsilon_{m} )(\varepsilon_{1} + 2\varepsilon_{2} ) + f_{1} (2\varepsilon_{2} - 2\varepsilon_{m} )(\varepsilon_{1} - \varepsilon_{2} )}},$$

where a_2_ is the outer radius, ε_1_ and ε_2_ are the core (of radius a_1_) and shell dielectric functions, respectively. The core volume fraction is $f_{1} = {{a_{1}^{3} } \mathord{\left/ {\vphantom {{a_{1}^{3} } {a_{2}^{3} }}} \right. \kern-\nulldelimiterspace} {a_{2}^{3} }}.$ It should be noted that the idea of effective susceptibility has been expressed for a long time (see, for example^23^, Chapt. 2) and as a result of the use of this idea can be indicated the obtaining of the Lorenz–Lorentz formula for the polarizability of the sphere in a homogeneous external field. Analogously ${\rm X}_{ij}^{(b)} ({\mathbf{R}},\omega )$ reflects the self-action processes for the particles located inside the homogeneous isotropic medium. But, $\Xi_{ij}^{(b)} ({\mathbf{R}},\omega )$ takes into account the self-action processes via small nanoparticle and, of course, depends on the distance between nanoparticles (see Fig. 1).

One should note that we have performed the self-consistency procedure when calculating the ponderomotive forces between nanospheres. The self-consistency was made in the frame of the method developed in^20^. A similar self-consistent procedure was demonstrated in^24^. But in the present work, we performed the self-consistency with the pseudo-vacuum Green function method and obtained the self-consistent equations for effective susceptibilities of the nanoparticles with taking into account the fact that nanoparticles are non-point-like and have their shape and dimensions, unlike in^24^ the same self-consistency was performed for the dipole moments of the nanoparticles in the dipole point-like approximation. Then the advantage of the present work is not only self-consistency but also the taking into account the inhomogeneities of local fields inside and at the nanoparticles.

In this regard, it is worth noting that the ponderomotive forces were calculated in the points of maximum gradients of local fields and, of course, the integrations of the density of the ponderomotive forces were over the volume of the shell of nanoparticle in the domains of hot spots of the local field.

For calculation of the local field intensity (including the ‘hot spots’), the equation of self-consistency (so-called Lippmann–Schwinger equation)^25^ should be used

4 $$\begin{aligned} E_{i} ({\mathbf{R}},\omega ) & = E_{i}^{(0)} ({\mathbf{R}},\omega ) \\ & \,\,\,\, - k_{o}^{2} \int\limits_{{V_{v} }} {d{\mathbf{R}}^{^{\prime}} G_{ij}^{(m)} ({\mathbf{R}},{\mathbf{R}}^{^{\prime}} ,\omega )\Xi_{jl}^{(v)} ({\mathbf{R}}^{^{\prime}} ,\omega )E_{l}^{(0)} ({\mathbf{R}}^{^{\prime}} ,\omega )} . \\ \end{aligned}$$

Then, the force density (the force per unit of volume) acting on the big nanoparticle can be written as

5 $$F_{i} ({\mathbf{R}}) = \left( {P_{j} ({\mathbf{R}}) \cdot \frac{\partial }{{\partial x_{j} }}} \right)E_{i} ({\mathbf{R}}),$$

where $P_{j} ({\mathbf{R}}) = \varepsilon_{0} \Xi_{jl}^{(v)} ({\mathbf{R}})E_{l}^{0} ({\mathbf{R}})$ is the polarization (local dipole moment) of the nanoparticle shell.

Since in formula (5) both the polarization and the field are local characteristics, and the polarization is distributed continuously over the shell of a large particle, the force acting on an element of the volume of the shell is calculated as the integral of (5) over this volume. In this sense, we are talking about the “density” of the force. It means that we calculate the force acting on the small volume of the nanoparticle for different areas of this nanoparticle. And since the force (5) is determined by the gradient of the local field, when calculating the normal and tangential components of the force acting on an element of the shell volume, we choose such an element that corresponds to the maximum value of the field gradient (hot spot area).

For example, Eq. (5) is equal to the equation of the force density in Eq. (3.62) of^26^. If the dipole moment density, i.e., the polarization, is replaced by the dipole moment, then Eq. (5) is a force that is equal to the first term of Eq. (2.2) of^27^. Then, using (4) we obtain

$$\begin{gathered} F_{i} ({\mathbf{R}}) = \left( {\varepsilon_{0} \Xi_{jk}^{(v)} ({\mathbf{R}})E_{k}^{0} ({\mathbf{R}}) \cdot \frac{\partial }{{\partial x_{j} }}} \right) \cdot \hfill \\ \left[ {E_{i}^{(0)} ({\mathbf{R}},\omega ) - k_{o}^{2} \int\limits_{{V_{v} }} {d{\mathbf{R}}^{^{\prime}} G_{is}^{(m)} ({\mathbf{R}},{\mathbf{R}}^{^{\prime}} ,\omega )\Xi_{sl}^{(v)} ({\mathbf{R}}^{^{\prime}} ,\omega )E_{l}^{(0)} ({\mathbf{R}}^{^{\prime}} ,\omega )} } \right]. \hfill \\ \end{gathered}$$

Taking into account that at the distances about the characteristic linear dimension of the nanoparticle, the external field is rather constant, this equation may be reduced to the following one

6 $$\begin{gathered} F_{i} ({\mathbf{R}}) = - k_{o}^{2} \varepsilon_{0} \Xi_{jk}^{(v)} ({\mathbf{R}})E_{k}^{0} ({\mathbf{R}}) \cdot \hfill \\ \int\limits_{{V_{v} }} {d{\mathbf{R}}^{^{\prime}} \frac{\partial }{{\partial x_{j} }}G_{is}^{(m)} ({\mathbf{R}},{\mathbf{R}}^{^{\prime}} ,\omega )\Xi_{sl}^{(v)} ({\mathbf{R}}^{^{\prime}} ,\omega )E_{l}^{(0)} ({\mathbf{R}}^{^{\prime}} ,\omega ).} \hfill \\ \end{gathered}$$

Note, in (6) the differentiation is over the components of ${\mathbf{R}}$. The action of the force onto the unit of the shell surface obviously can change the shell properties up to its destruction.

In the model of ellipsoid susceptibility, homogeneous field tensor $\Xi_{jk}^{(v)} ({\mathbf{R}})$ does not depend on coordinate^23,24^, i.e. $\Xi_{jk}^{(v)} ({\mathbf{R}}) \to \Xi_{jk}^{(v)}$. Then, the dependence of the force from (6) on coordinate has its origin from the local field distribution at the surface of the big nanoparticle only—via the dependence of the Green function $G_{is}^{(m)} ({\mathbf{R}},{\mathbf{R^{\prime}}},\omega )$ on **R** [see (6)]. Remember that the external field $E_{l}^{(0)} ({\mathbf{R}},\omega )$ is the constant in the near-field approximation. Then it can be obtained from (6)

7 $$F_{i} ({\mathbf{R}}) = - k_{o}^{2} \left( {\varepsilon_{0} \Xi_{jk}^{(v)} \cdot \int\limits_{{V_{v} }} {d{\mathbf{R}}^{^{\prime}} \frac{\partial }{{\partial x_{j} }}G_{is}^{(m)} ({\mathbf{R}},{\mathbf{R}}^{^{\prime}} ,\omega )\Xi_{sl}^{(v)} } } \right)E_{k}^{(0)} E_{l}^{(0)} .$$

As the ‘hot spots’ (the domains of strong local field) locations are defined by the Green function dependence on **R**, the forces acting on the big (shelled) nanoparticle will be concentrated at the hot spots (Fig. 3c).

## Results of numerical calculations

**Figure 2:**
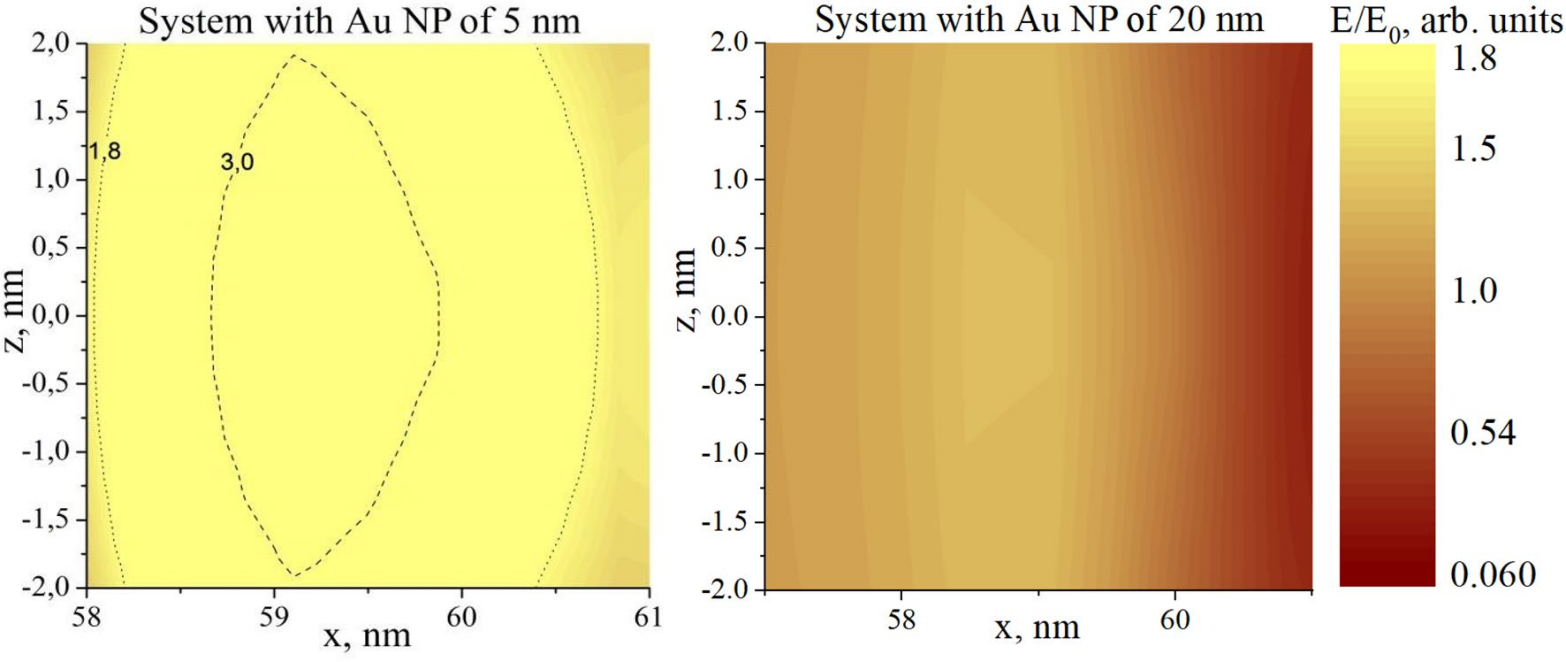
Distribution of local field in the system of the nanoparticle of 120 nm diameter with 10 nm shell ($\varepsilon_{core} = 2$, $\varepsilon_{shell} = 4$) located close to the nanoparticle of 5 nm diameter (left) and of 20 nm diameter (right) with $\varepsilon_{np} = - 10.5 + 1.3i$ in the medium with $\varepsilon_{m} = 1$, $E = 1\; {\rm V}/{\rm m}$. The section at the $y = 0$ (dotted line is the surface of the bigger nanoparticle). Note, for the system with the smaller nanoparticle the field is much higher than 1.8. This area is shown by lines.

In this section, we represent the results of calculations of the mechanical force distribution, which appears in the system of two different-sized nanoparticles. As it was mentioned previously,

the force is caused by the action of the local field gradient on the polarized object. Hence, the force gains its maximum values at the points of the highest field gradient. Typical local field distribution for the system of two nanoparticles is shown in Fig. 2, others can be found e.g. in^25,26^.

It can be seen that the ‘hot spots’ and highest gradients are located at the surface of the biggest nanoparticle. Hence, we need to calculate the distribution of tangential and normal forces at the surface of the biggest nanoparticle. Such presentation of results is convenient for their analysis and understanding. In the following calculations we set the distance between the surfaces of the nanoparticles equal to the diameter of the smaller nanoparticle (i.e. 5 and 20 nm).

**Figure 3:**
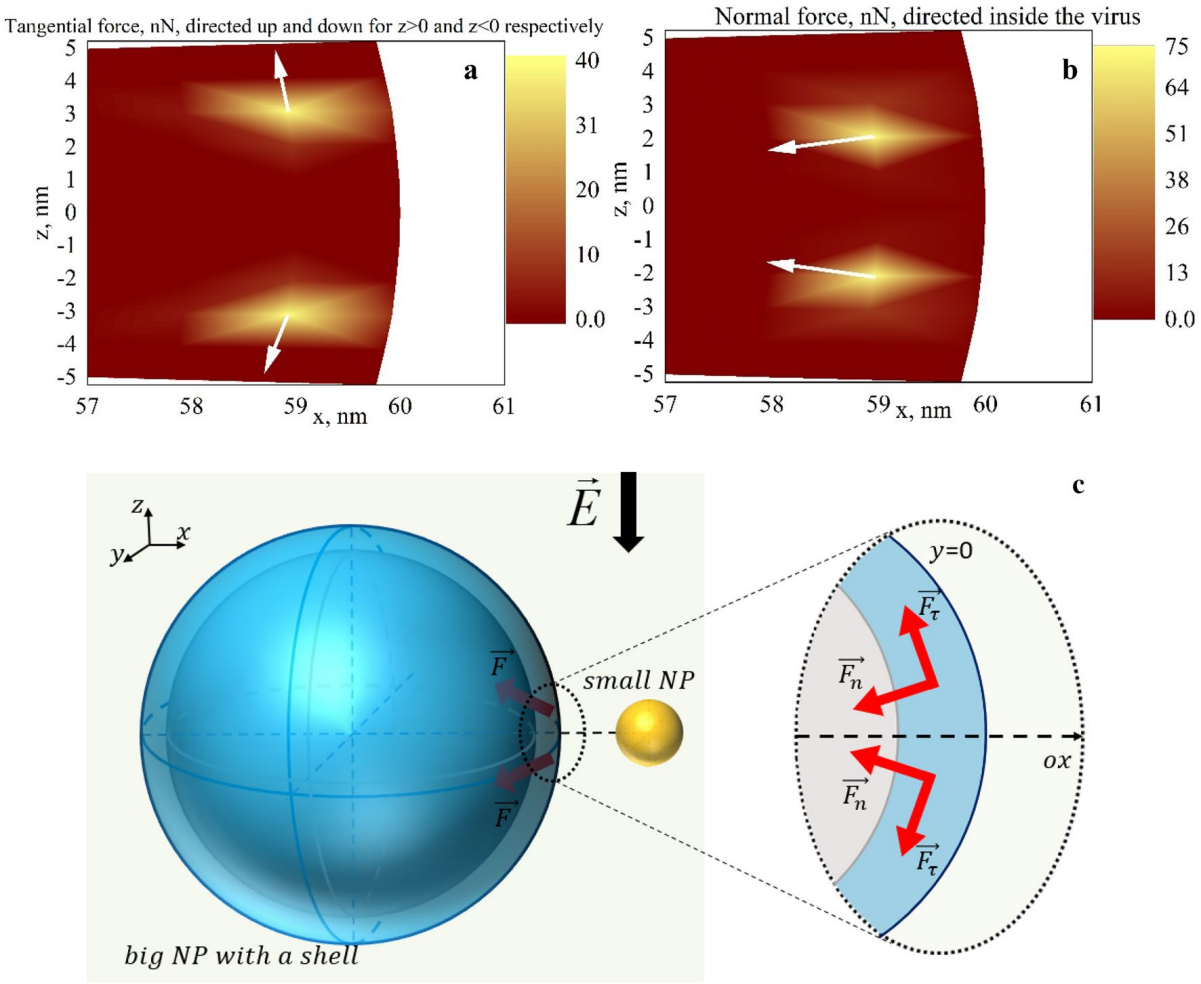
Distribution of tangential (**a**) and normal force (**b**) density on the surface of the nanoparticle of 60 nm radius with 10 nm shell ($\varepsilon_{core} = 2$, $\varepsilon_{shell} = 4$) located close to the nanoparticle of 2.5 nm radius with $\varepsilon_{np} = - 10.5 + 1.3i$ in the medium with $\varepsilon_{m} = 1$,$\lambda = 615 {\rm nm}$, $E = 1\; {\rm V}/{\rm m}$ (The section at the $y = 0$). (**c**)—Sketch of the forces acted on the bigger nanoparticle in the system of two different-sized nanoparticles.

As the main goal of the work is to reveal the possible presence of mechanical forces in the system of two nanoparticles, we conducted calculations for one system with the following parameters: the bigger nanoparticle is of 120 nm diameter with a shell of 10 nm thickness and the smaller nanoparticle is of 5 nm diameter without a shell. The dielectric constant of the bigger nanoparticle core is 2, the one of the nanoparticle shell is 4, and the one of the smaller nanoparticl is $- 10.5 + 1.3i$. The system is supposed to be located in the medium with $\varepsilon_{m} = 1$, $E = 1\; {\rm V}/{\rm m}$. The results of the calculations are presented in Fig. 3.

It can be seen, that indeed in the system the strong mechanical forces may appear close to the surface of the bigger nanoparticle due to the local field redistribution and the nanoparticle polarization. However, the field of force is quite narrowly localized, which can be easily understood from the local field distribution described previously. The interesting result is that the forces are directed inside the bigger nanoparticle on different sides. Such force deforms the nanoparticle and may lead to its breakage when the stress caused by the force prevails the nanoparticle limit of strength.

**Figure 4:**
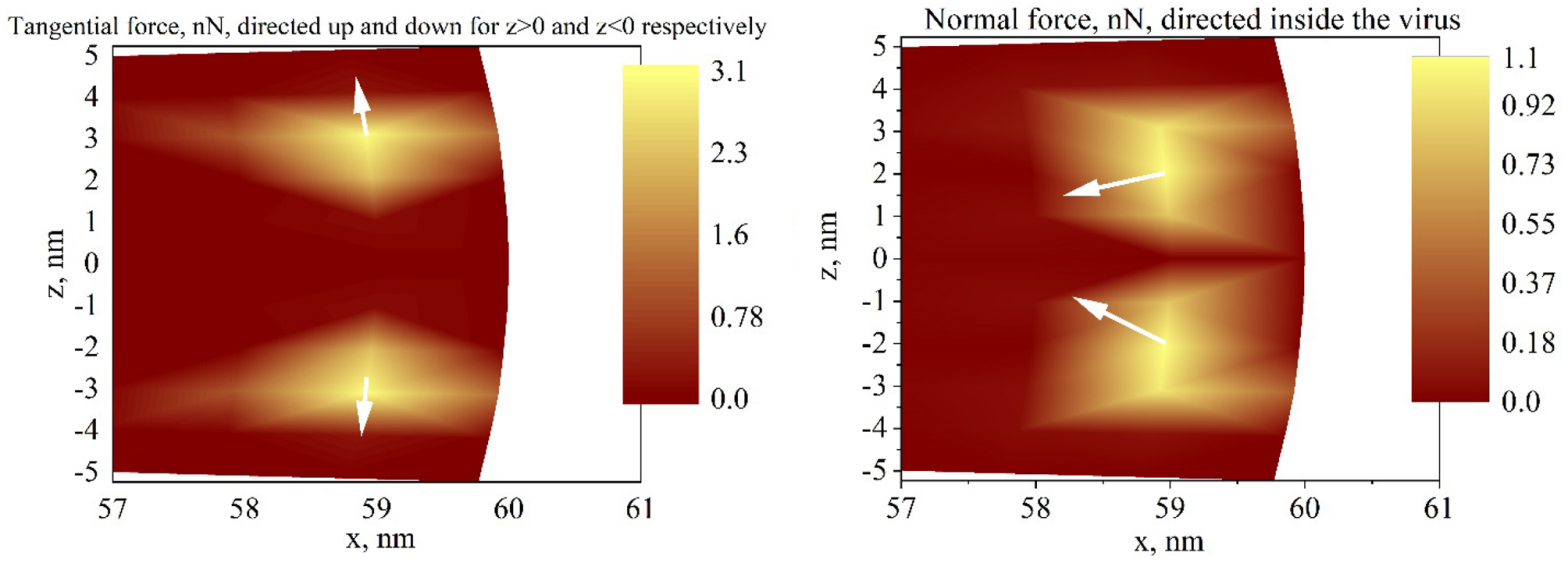
Distribution of tangential (left) and normal force (right) density on the surface of the nanoparticle of 60 nm radius with 10 nm shell ($\varepsilon_{core} = 2$, $\varepsilon_{shell} = 4$) located close to the nanoparticle of 10 nm radius with $\varepsilon_{np} = - 10.5 + 1.3i$ in the medium with $\varepsilon_{m} = 1$, $\lambda = 615\,{\rm nm}$. The section at the $y = 0$.

As it was mentioned above, the nanoparticle’s size and shape are the main parameters that influence the local field distribution. However, for the non-spherical nanoparticles, the model should be much more complicated. But we can apply the same model for the system with nanoparticles of bigger sizes^27,28^. Results for a similar system with the diameter of the smaller nanoparticle of 20 nm are presented in Fig. 4. Note, that the maximum force is about one order of magnitude smaller compared to the system with the smaller nanoparticle in Fig. 2. These results are in good coincidence with previous calculations and well-known experimental facts, that the local field enhancement effect is much stronger near smaller nanoparticles.

## Discussion: interaction between the virus and nanoparticle

One of the interesting possible applications of the described effect may be found in nanomedicine. Recently numerous studies on the interaction between viruses and nanoparticles are provided because of the interest to develop new methods of antiviral therapy. These studies have shown, that the interaction between the nanoparticles and viruses can be the reason for the loss by the virus of its infectivity^27–33^. The effect is observed equally well for different types of nanoparticles, which excludes the chemical reasons for the effect. As the domains of high field mean the existence of gradients of the local field at the surface of bigger nanoparticles, i.e. they are localized at the virus shell. The molecules at the surface of the virus contain the polar sites^34,35^. Consequently, the dipole moments of the polar sites are under the action of field gradients. It means that the ponderomotive forces acting on these molecules arise. Hence, in such systems, the virus shell deformation or destruction may appear. This effect was demonstrated, in particular, for interaction between the TiO_2_ nanoparticles and influenza viruses H1N1 type when the viral envelope was destructed for 15 min of exposition^30^. In the work^28^ numerous results of the action of nanoparticles on the viruses were analyzed and it was concluded that the interaction between nanoparticles and viruses has a field nature and can be connected with the local-field enhancement effect. The results of similar experiments provided for the system of gold nanoparticles of different sizes and influenza virus H1N1 were reported in our previous work^31^. As in all mentioned works, the antiviral effect is reported to be higher for the smaller nanoparticles, it can be supposed that smaller nanoparticles due to higher forces may reshape or even destroy the virus. On the other side, the use of nanoparticles (especially nanoparticles of heavy metals) in antiviral therapy faces the problem of their removal (or disposal) from a living organism. It has been suggested, and previously tested in Ref.^36^, that complex nanoparticles, such as a gold nanoparticle in a silica shell or a gold nanoparticle located on the surface of a larger silica nanoparticle, can be excreted from a living organism. It should be noted that there are a large number of works discussing the use of various nanoparticles with shells in biology (see, for example, works^37–39^). And in this regard, there is a need to calculate the effect of ponderomotive forces on the virus, when the virus interacts with complex nanoparticles. Hence, the proposed method may be useful for solving the mentioned problems. More detailed both experimental and theoretical investigations of virus-nanoparticle interactions will be conducted in further works.

## Conclusions

The local field enhancement effect and physical adsorption in nanosystems due to Van der Waals interaction are well-known phenomena in nanophysics. The model and calculations presented in the work show that as a consequence of such electromagnetic processes the ponderomotive forces acting on the nanoparticle appear. It is obvious, that these forces need to be taken into account as they may lead to deformation or even destruction of nanoparticles in nanosystems. As it can be seen from the model, the forces depend on the local field gradient, which in turn depends on the sizes, shapes, and materials of nanoparticles^21^. For example, as in systems with bigger nanoparticles, the local field gradient is lower^23^, and the values of the forces in these systems are lower too.

## Supplementary Information

Supplementary Information.

## Data Availability

The datasets used and/or analyzed during the current study are available from the corresponding author on reasonable request.
