# Supplementary material for: Ponderomotive forces in the system of two nanoparticles
Source: Sci Rep. 2022 Oct 22;12:17768. doi: 10.1038/s41598-022-22510-8 (PMC9588014; doi:10.1038/s41598-022-22510-8)
Supplement: Supplementary file 1 — Supplementary Information. [file 41598_2022_22510_MOESM1_ESM.docx]

**Appendix.** Pseudo-vacuum Green function.

Consider a single nanoparticle type 1 or type 2 embedded in the medium (0) with dielectric constant ε_m_ . The linear response to long-range external field can be calculated in the frame of approach developed in Ref.[15]. The approach takes into account the self-action effects (Fig.1A). Then the effective susceptibility is the characteristic of nano-object depended on dimension, shape and material of which the nano-object is fabricated. Calculation of the effective susceptibility of nanoparticle of type 1 gives Eq.(3). Calculation of effective susceptibility of nanoparticles type 2 gives [15]

Fig.1A. Sketch of formation of effective susceptibility of a single nanoparticles of type 1 (shelled nanoparticle) and type 2 (golden nanoparticle of dimension about of few nanometers) in the homo-geneous and isotropic medium charac-terized by dielectric constant ε_m_

$X_{ij}^{(p)}(\mathbf{R},\omega)=\left[\left(\chi_{p}\delta_{ij}\right)^{-1}-k_{0}^{2}\int_{V_{v}} d\mathbf{R}'G_{ji}^{(0)}(\mathbf{R},\mathbf{R}',\omega)\right]^{-1}$, (1A)

where $\chi_{p}=\varepsilon_{p}-\varepsilon_{m}$ , ε_p_ is the dielectric constant of material of which the nanoparticle is fabricated, and Green function $G_{ij}^{(0)}(\mathbf{R},\mathbf{R}',\omega)$ describes the electrodynamic properties of homogeneous isotropic medium with dielectric constant ε_m_ , integration is over the volume of nanoparticle. For spherical nanoparticle one obtains well known expression (Lorenz-Lorentz relation) as it was shown in Ref.[37]

$X_{ij}^{(p)}(\omega)=X^{(p)}(\omega)\delta_{ij},\text{ }X^{(p)}(\omega)=4\pi\varepsilon_{0}R_{p}^{3}\frac{\varepsilon_{p}-\varepsilon_{m}}{\varepsilon_{p}+2\varepsilon_{m}}$, (2A)

with $R_{p}$radii of the nanoparticle and. As was mentioned above, the electrodynamic properties of the medium are described by Green function (photon propagator) $G_{ij}^{(0)}(\mathbf{R},\mathbf{R}',\omega)$. To obtain the electrodynamic Green function of the system ‘nanoparticle-medium’ we should take into account the processes of light scattering inside the system. Consider the system ‘nanoparticle inside the medium’. Let the external field induced by point-like dipole located at the point **R**’ $P_{j}(\mathbf{R}''-\mathbf{R}',\omega)=P_{j}^{(0)}(\omega)\delta(\mathbf{R}'''-\mathbf{R}')$acts to the system. Then, the electric field at the any point **R** consists of the external field

$E_{i}^{(0)}(\mathbf{R},\omega)=\int_{V} d\mathbf{R}''G_{ij}^{(0)}(\mathbf{R},\mathbf{R}'',\omega)P_{j}^{(0)}\delta(\mathbf{R}''-\mathbf{R}')=G_{ij}^{(0)}(\mathbf{R},\mathbf{R}',\omega)P_{j}^{(0)}(\omega)$ , (3A)

and the field scattered by nanoparticle (see Fig.2A)

Fig.2A. Sketch of formation of the total field in the system ‘nanoparticle+medium’ to obtain the pseudo-vacuum Green function

$\begin{matrix} E_{i}^{(Sc)}(\mathbf{R},\omega)=-k_{0}^{2}\int_{V_{p}} d\mathbf{R}''G_{il}^{(0)}(\mathbf{R},\mathbf{R}'',\omega)X_{lk}^{(p)}(\omega)E_{k}^{(0)}(\mathbf{R}'',\omega)= \\ \text{ }=-k_{0}^{2}\int_{V_{p}} d\mathbf{R}''G_{il}^{(0)}(\mathbf{R},\mathbf{R}'',\omega)X_{lk}^{(p)}(\omega)G_{kj}^{(0)}(\mathbf{R}'',\mathbf{R}',\omega)P_{j}^{(0)}(\omega). \end{matrix}$ (4A)

Superposition of the fields principle gives the total electric field at the point **R**

$E_{i}(\mathbf{R},\omega)=E_{i}^{(0)}(\mathbf{R},\omega)+E_{i}^{(Sc)}(\mathbf{R},\omega)$ . (5A)

Substitution of Eqs.(3A) and (4A) into Eq.(5A) gives

$E_{i}(\mathbf{R},\omega)=\left[G_{ij}^{(0)}(\mathbf{R},\mathbf{R}',\omega)-k_{0}^{2}\int_{V_{p}} d\mathbf{R}''G_{il}^{(0)}(\mathbf{R},\mathbf{R}'',\omega)X_{lk}^{(p)}(\omega)G_{kj}^{(0)}(\mathbf{R}'',\mathbf{R}',\omega)\right]P_{j}^{(0)}(\omega)$ . (6A)

The expression inside the brackets obviously is the Green function $G_{ij}^{(m)}(\mathbf{R},\mathbf{R}',\omega)$of ‘new medium’ consisting of the medium ‘0’ and nanoparticle. The approach demonstrated above was proposed by O.Keller [16] and obtained Green is usually named as pseudo-vacuum Green function. Note, all number scatterings were taken into account in this approach. It was demonstrated, for example, in Ref.[37] by summation of infinite number diagrams (in the frame of Feynman diagram method).
